# The “Aging Factor” Eotaxin-1 (CCL11) Is Detectable in Transfusion Blood Products and Increases with the Donor’s Age

**DOI:** 10.3389/fnagi.2017.00402

**Published:** 2017-12-01

**Authors:** Julia Hoefer, Markus Luger, Christian Dal-Pont, Zoran Culig, Harald Schennach, Stefan Jochberger

**Affiliations:** ^1^Experimental Urology, Department of Urology, Medical University of Innsbruck, Innsbruck, Austria; ^2^Department of Anesthesiology and Critical Care Medicine, University Hospital of Innsbruck, Innsbruck, Austria; ^3^Central Institute for Blood Transfusion and Immunological Department, University Hospital of Innsbruck, Innsbruck, Austria

**Keywords:** eotaxin-1, aging, cognitive function, blood components, blood transfusion, age

## Abstract

**Background**: High blood levels of the chemokine eotaxin-1 (CCL11) have recently been associated with aging and dementia, as well as impaired memory and learning in humans. Importantly, eotaxin-1 was shown to pass the blood-brain-barrier (BBB) and has been identified as crucial mediator of decreased neurogenesis and cognitive impairment in young mice after being surgically connected to the vessel system of old animals in a parabiosis model. It thus has to be assumed that differences in eotaxin-1 levels between blood donors and recipients might influence cognitive functions also in humans. However, it is unknown if eotaxin-1 is stable during processing and storage of transfusion blood components. This study assesses eotaxin-1 concentrations in fresh-frozen plasma (FFP), erythrocyte concentrate (EC), and platelet concentrate (PC) in dependence of storage time as well as the donor’s age and gender.

**Methods**: Eotaxin-1 was measured in FFP (*n* = 168), EC (*n* = 160) and PC (*n* = 8) ready-to-use for transfusion employing a Q-Plex immunoassay for eotaxin-1. Absolute quantification of eotaxin-1 was performed with Q-view software.

**Results**: Eotaxin-1 was consistently detected at a physiological level in FFP and EC but not PC. Eotaxin-1 levels were comparable in male and female donors but increased significantly with rising age of donors in both, FFP and EC. Furthermore, eotaxin-1 was not influenced by storage time of either blood component. Finally, eotaxin-1 is subject to only minor fluctuations within one donor over a longer period of time.

**Conclusion**: Eotaxin-1 is detectable and stable in FFP and EC and increases with donor’s age. Considering the presumed involvement in aging and cognitive malfunction, differences in donor- and recipient eotaxin-1 levels might affect mental factors after blood transfusion.

## Introduction

Eotaxin-1, (also known as CC-motif chemokine ligand 11, CCL11), is a chemokine that acts as selective chemo-attractant for eosinophil granulocytes (Matthews et al., 1998) and thus is involved in multiple eosinophil-associated diseases such as atopic dermatitis, allergic rhinitis, sinusitis, asthma, ulcerative colitis as well as in the response to parasitic infections (Chao et al., 2012; Geiger et al., 2013; Waddell et al., 2013). However, eotaxin-1 has also been implicated in prostate- and ovarian cancer (Levina et al., 2009; Nolen and Lokshin, 2010; Agarwal et al., 2013; Zhu F. et al., 2014; Heidegger et al., 2015), as well as in vascular inflammation and atherosclerosis in a non-eosinophil dependent manner (Haley et al., 2000; Zee et al., 2004). Interestingly, chemokines such as eotaxin-1 have also been suggested to act as neuromodulators (Rostene et al., 2007). These findings prove that eotaxin-1 exerts manifold biological functions in multiple tissues and organs.

It is known that some patients experience cognitive or neurological disturbances such as postoperative cognitive dysfunctions (POCD) after major surgery, especially when having received blood transfusions (Shiraboina et al., 2014; Zhu S.-H. et al., 2014). POCD is characterized by transient or permanent confusion, mental slowing, and cognitive impairments regarding memory, verbal abilities, perception, attention, executive functions, abstract thinking and others (Deiner and Silverstein, 2009; Monk and Price, 2011), conditions which are also observed during normal aging (Simen et al., 2011). Although the responsible mechanisms are not completely clear, POCD has recently been associated with enhanced inflammatory processes in the brain (Terrando et al., 2011) which might be caused by yet unknown, blood-borne “aging factors”.

In this context, Villeda et al. (2011) demonstrated that Eotaxin-1 is associated with aging and cognitive functions. Eotaxin-1 was increased in aged mice as well as in young mice after being connected to the blood flow of aged mice. Strikingly, eotaxin-1 levels correlated most strongly with reduced neurogenesis and impaired hippocampal related memory and learning in these parabiosis models. As a proof of concept, the group further showed that artificially increasing eotaxin-1 levels in young mice was sufficient to resemble the cognitive impairments observed in old animals (Villeda et al., 2011). Furthermore, eotaxin-1 negatively correlated with memory function in Alzheimer’s disease patients (Bettcher et al., 2016). Importantly, it has recently been demonstrated that eotaxin-1 is able to pass the blood-brain-barrier (BBB; Erickson et al., 2014), which enables the chemokine to exert direct effects in the brain. Taken together, despite lacking an exact mechanistic explanation, the blood-borne CCL11 is highly suspected of affecting neurogenesis, cognitive functions and aging in the adult brain of mice, but also humans. Thus, it is possible that transfusion of blood products from donors with high eotaxin-1 levels might elevate total eotaxin-1 levels in recipients, with implications on cognitive capabilities. However, to the best of our knowledge it has not been elucidated if and to which extent eotaxin-1 is present in different blood components stored for transfusions, and whether it can be reliably measured in these fluids. Therefore, the present study aims to investigate if erythrocyte concentrate (EC), platelet concentrate (PC), and fresh-frozen plasma (FFP) contains measurable eotaxin-1, if eotaxin-1 concentrations resemble those found in unprocessed blood components, and if eotaxin-1 levels in transfused blood products correlate with the donor’s age or gender, as well as the storage time of the blood products.

## Materials and Methods

All experiments have been conducted in accordance with the Helsinki Declaration after having obtained permission from the Institutional Ethics Review Board of Medical University of Innsbruck (Number: AN2014-0165 337/4.11). All blood donors gave written informed consent in accordance with the declaration of Helsinki.

### Samples

Leukocyte depleted ready- to-use blood products from volunteer donors (FFP, [*n* = 168], EC [*n* = 160], and PC [*n* = 8]) were used. All blood products have been processed and stored for later transfusion at the Central Institute for Blood Transfusion and Immunological Department of the University Hospital Innsbruck, Austria.

### Volunteer Donors

At the University Hospital of Innsbruck, volunteer blood donors must fulfill stringent, standardized health criteria. Before donation, all persons have to fill in medical questionnaires regarding general health status, recent diseases or medication or recent traveling. After that, a physical examination including measurement of blood pressure, heart rate, temperature and hemoglobin concentration is performed. The donated blood is further tested for neopterin as well as HIV, hepatitis, syphilis and parvovirus B19 infections. If any disease or abnormality is detected, the donated blood is discarded. Based on this procedure, it can be assumed that all donors have been in good health at the time of blood donation. Donors’ characteristics (age range, gender, blood type) are summarized in Supplementary Figure S1.

### Preparation of EC

Leukocyte depleted ECs are produced from single-donor fresh whole blood by cell-separation. One hundred milliliters of additive solution (SAGM) is added in order to compensate for plasma and nutrients. Each 280 ml EC package exhibits a hematocrit between 50 and 70 and contains less than 1*10^6^ leukocytes. ECs are stored for a maximum of 42 days at 4°C. Storage time of used ECs ranged from 4 days to 29 days.

### Preparation of FFP

Leukocyte depleted FFPs are won from single-donor full-blood by cell separation and apheresis. FFPs can be stored for a maximum of 2 years at −30°C. Storage time of used FFPs ranged from 7 days to 76 days. For determination of eotaxin-1 variability within 1 donor over time, 10 ml blood samples from one donor drawn at 11 different days during 3 months, in part at multiple times a day, resulting in a total of 15 samples. These samples underwent the same processing as FFP samples.

### Preparation of PC

Leukocyte depleted PCs from single donors are prepared by thrombocyte-apheresis. Each 300 ml PC is composed of 2–4 × 10^11^ platelets, suspended in plasma additive solution. For safety reasons all PC are pathogen inactivated by amotosalen/UVA treatment (INTERCEPT blood system, CERUS corporation). PCs are packed in gas permeable plastic bags and stored at room temperature (20–24°C) under continuous movement for a maximum of 7 days. Storage time of all used PCs was 2 days.

### Sampling and Eotaxin-1 Measurement

FFP, EC and PC samples for the present study have been obtained by shrink-wrapping a small amount (2 ml) from the whole blood product and were stored under the respective standard conditions until being subjected to measurement. A Q-Plex immunoassay for infrared (IR) based detection of human eotaxin-1 (Quansys Biosciences) was used according to the manufacturer’s instructions. All samples were measured in duplicates. Signals were detected using Odyssey infrared scanner (LI-COR Biosciences) and eotaxin-1 was quantified with Q-View Software (Quansys) in relation to an 8-point eotaxin-1 standard curve (3500 pg/ml to 4.8 pg/ml).

### Statistical Methods

SPSS and GraphPad Prism 5 were used for statistical analyses. Gaussian distribution was not given, as determined by Kolmogorov-Smirnov normality test. Differences between groups were analyzed using Mann–Whitney test (when comparing two groups) or non-parametric Kruskal-Wallis one way analysis of variance (ANOVA) test including Dunn’s *post hoc* analysis (when comparing more than two groups). All differences highlighted by asterisks were statistically significant as encoded in figures (*p < 0.05, **p < 0.01, ***p < 0.001). Data are presented as Box-Whiskers Plots showing median and 10–90 percentiles, unless otherwise stated.

## Results

### Eotaxin-1 Is Detectable in Donated Blood Products and Independent of Storage Time

**Figure 1:**
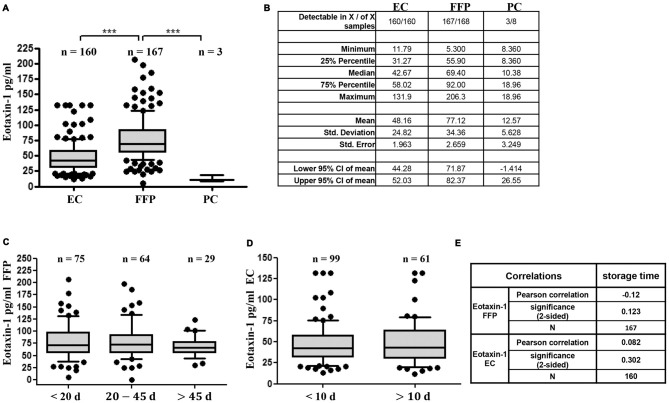
Eotaxin-1 is detectable and stable in fresh-frozen plasma (FFP) and erythrocyte concentrate (EC), but not in platelet concentrate (PC). **(A,B)** Eotaxin-1 was measured in EC, FFP and PC. **(A)** Data are presented as Box-Whiskers Plot, showing median and 10–90 percentiles. Statistical analysis was performed using Kruskal-Wallis one way analysis of variance (ANOVA) test including Dunn’s *post hoc* analysis (***p < 0.001). **(B)** Tabular summary and column statistics of EC, FFP, PC samples analyzed in **(A)**. **(C,D)** Eotaxin-1 was measured in **(C)** FFP and **(D)** EC and analyzed in dependence of storage time. Data are presented as Box-Whiskers Plot, showing median and 10–90 percentiles. **(E)** Pearson correlation analysis of eotaxin-1 in FFP and EC with storage time.

First, we assessed eotaxin-1 levels in different blood components ready to be transfused to patients using a custom-made Q-Plex immunoassay of human eotaxin-1. While eotaxin-1 was detectable only in three out of eight PC (median: 10.4 pg/ml; range: 8.4–19), we found measurable eotaxin-1 in 167 of 168 fresh frozen plasma (FFP) and in 160 of 160 EC samples. Eotaxin-1 levels were consistently higher in FFP (median: 69.4 pg/ml; range: 5.3–206.3) compared to EC (median: 42.7 pg/ml; range: 11.8–131.9; Figures 1A,B). It has been suggested, that cytokines or chemokines might accumulate or decrease over time in stored blood products (Zimring, 2013; Corsi et al., 2014; Aziz et al., 2016). To assess eotaxin-1 stability in processed blood products, we next analyzed eotaxin-1 in FFP and EC in relation to storage time. All samples used in the present study have been stored for 7–76 days (FFP; −20°C) or 4–29 days (EC; 4°C) after processing. As shown in Figures 1C–E, storage time does not correlate with the measured eotaxin-1 levels, neither in FFP nor in EC.

### Eotaxin-1 Correlates with Age but Does Not Differ between Male and Female Donors

**Figure 2:**
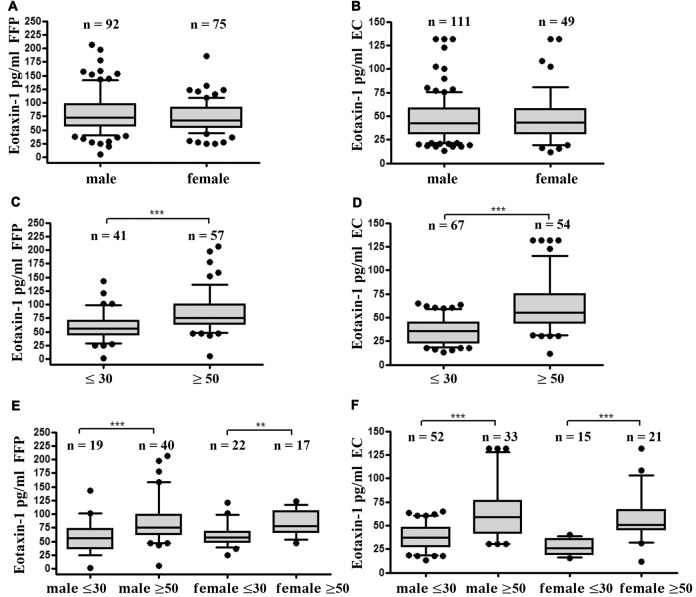
Eotaxin-1 is comparable in male and female donors but is increased in older donors. **(A,B)** Eotaxin-1 was measured in **(A)** FFP and **(B)** EC and analyzed in dependence of gender. Data are presented as Box-Whiskers Plot, showing median and 10–90 percentiles. **(C,D)** Eotaxin-1 was measured in **(C)** FFP and **(D)** EC and analyzed in dependence of age. Data are presented as Box-Whiskers Plot, showing median and 10–90 percentiles. Statistical analysis was performed using Mann-Whitney U test (***p < 0.001). **(E,F)** Eotaxin-1 was measured in **(E)** FFP and** (F)** EC and analyzed in dependence of age and gender. Data are presented as Box-Whiskers Plot, showing median and 10–90 percentiles. Statistical analysis was performed using Kruskal-Wallis one way ANOVA test including Dunn’s *post hoc* analysis (**p < 0.01; ***p < 0.001).

**Figure 3:**
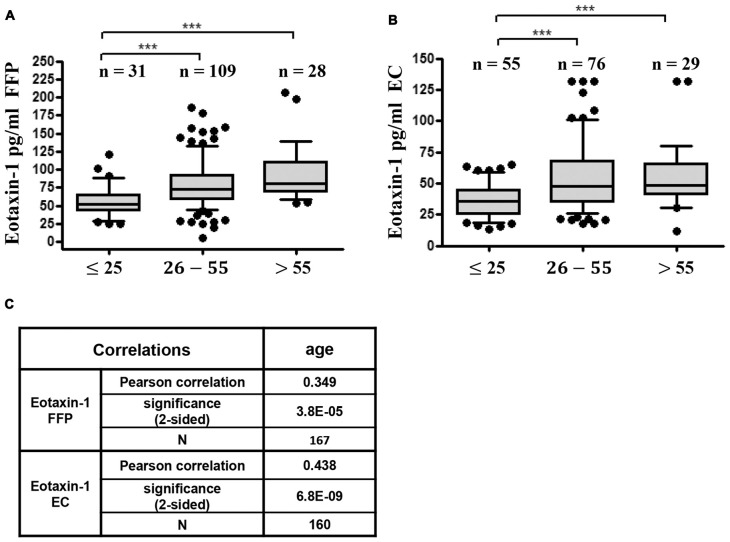
Eotaxin-1 correlates with age.** (A,B)** Eotaxin-1 was measured in **(A)** FFP and** (B)** EC and analyzed in dependence of age. Data are presented as Box-Whiskers Plot, showing median and 10–90 percentiles. Statistical analysis was performed using Kruskal-Wallis one way ANOVA test including Dunn’s *post hoc* analysis (***p < 0.001). **(C)** Pearson correlation analysis of eotaxin-1 in FFP and EC with age.

As a next step we investigated gender- and age specific differences in eotaxin-1 expression in FFP and EC. Eotaxin-1 levels were equally distributed in male and female donors in both blood components (Figures 2A,B). Interestingly, both in FFP and EC eotaxin-1 levels were significantly higher in old (>50 years) than in young (<30 years) donors (Figures 2C,D), and this effect was similar in both sexes (Figures 2E,F). A more detailed age specific analysis revealed that eotaxin-1 specifically increases in middle-aged persons, given that the chemokine is significantly elevated in middle-aged (26–55) compared to very young (18–25) donors, and only tends to further increase in donors above the age of 55 years (Figures 3A,B). Consistently, correlation analysis revealed a significant positive correlation of eotaxin-1 and age in both blood products (Figure 3C).

### Eotaxin-1 Level Is Stable within One Donor during Different Time Points

**Figure 4:**
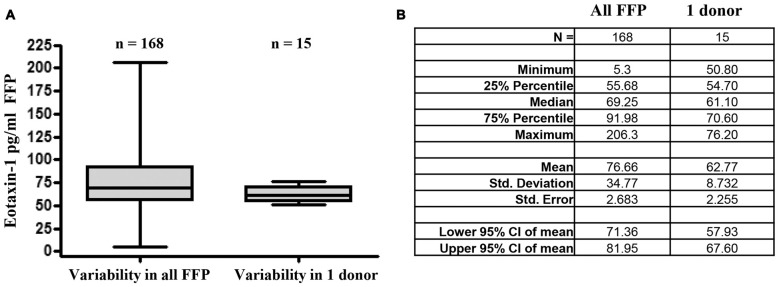
Eotaxin-1 is subject to minor fluctuations within one donor. **(A)** Eotaxin-1 was measured in FFP of 160 donors, or 15 plasma samples from one volunteer drawn at different time points within a period of 30 days. Plasma has been processed using the same protocol as for FFP. Data are presented as Box-Whiskers Plot showing median, minimum and maximum. **(B)** Tabular summary and column statistics of samples analyzed in **(A)**.

In order to estimate the variability of eotaxin-1 within one individual over time, we assessed eotaxin-1 levels in 15 blood samples obtained from one volunteer during a period of 3 months (Figures 4A,B). For comparability, these samples have been processed following the protocol for FFP production. Of note, eotaxin-1 in plasma from one donor varied only to a minor extent (median: 61.1; range: 50.8–70.6), when compared to the overall variability within all FFPs (median: 69.3 pg/ml; range: 5.3–206.3).

## Discussion

Recently, high plasma eotaxin-1 has been associated with impaired cognitive functions such as memory and learning, and eotaxin-1 has been found to correlate with dementia in Alzheimer’s disease patients (Bettcher et al., 2016). In this study, we show for the first time that ready-to-use transfusion blood components contain the CCL11 at a physiological concentration. Eotaxin-1 was detected in 160/160 EC (median 42.7 pg/ml) and 167/168 FFPs (median 69.4 pg/ml). Importantly, in both blood components eotaxin-1 expression did not differ between males and females, but significantly increased with the donor’s age in both sexes. Of note, eotaxin-1 levels detected in these processed, transfusible blood products are comparable with those found in unprocessed plasma of healthy individuals (Villeda et al., 2011). In this context, Agalliu et al. (2013) report a median eotaxin-1 plasma concentration of ~70 pg/ml in men and ~40 pg/ml in women. Of note, the scope of that study was to assess reproducibility of cytokine-measurements and not to detect demographic differences in eotaxin-1. Therefore, by chance, male donors have been much older (average 62 years) than female donors (average 32 years). This fact may well explain the differential eotaxin-1 expression between male and female donors in their study which we did not detect in our analysis, and further underlines the finding that eotaxin-1 increases with age. Importantly, Agalliu et al. (2013) also proved an excellent intra-subject reproducibility when analyzing multiple samples from the same donor within three subsequent years, with a slight eotaxin-1 increase from year 1 to year 3 in one donor. In the present study, we were able to confirm this finding by demonstrating that eotaxin-1 is subject to only minor donor-specific changes over 15 measurements within a 3-months period of time, even when samples have been taken at different times of day. Taken together, our findings and the available literature prove that eotaxin-1 is a relatively stable factor in fresh or processed blood components of one individual over a longer period of time, but significantly rises with increasing age.

Importantly, Eotaxin-1 was found increased in Alzheimer’s disease patients compared to age-matched controls. Eotaxin-1 correlated with impaired verbal and visual memory (Bettcher et al., 2016), and other conditions associated with cognitive decline, such as recurrent depression (Jorm, 2000) have also been associated with increased levels of the chemokine (Grassi-Oliveira et al., 2012). Moreover, Villeda et al. (2011) demonstrated that, among a range of cytokines and chemokines, eotaxin-1 correlated most strongly with reduced hippocampal neurogenesis and aging in mice, and importantly, artificially increasing eotaxin-1 levels in young mice resulted in decreased neurogenesis as well as in impaired memory and learning (Villeda et al., 2011). Using mouse parabiosis models (i.e., two mice surgically joined to each other, leading to a vascular anastomosis) Villeda et al. (2011) further proved that exposure of a young animal to the systemic milieu of an older mouse is sufficient to induce severe cognitive impairments and reduced neurogenesis after 2–5 weeks, and identify blood-borne eotaxin-1 as one of the responsible factors. It is important to mention that eotaxin-1 has recently been shown to undergo bidirectional transport across the BBB through direct interaction with the BBB, as well as via binding to cellular blood components. While efflux from the brain was slow and saturable, influx was fast and resulted in eotaxin-1 accumulation in most brain areas tested (Erickson et al., 2014). The mechanisms of how eotaxin-1 affects brain function are currently not understood. On one hand, eotaxin-1 might promote eosinophil influx into the brain with subsequent neural damage. However, chemokines such as eotaxin-1 are suspected to act as neuromodulators also independently of their role as chemoattractants (Rostene et al., 2007). More precisely, eotaxin-1 is suspected to cause NOX1 upregulation, which induces neuronal death by promotion of migration and production of reactive oxygen species in microglia (Parajuli et al., 2015). Taken together, a growing body of evidence indicates that circulating eotaxin-1 passes the BBB and directly regulates multiple brain functions.

In the present study, we prove that eotaxin-1 is still detectable at a normal level in processed blood products ready for transfusion and its concentration greatly differs between donors in an age-dependent manner. Thus, transfusion of larger amounts of blood components from older donors to younger recipients may increase total eotaxin-1 blood levels of the recipient. Based on the above mentioned findings, it is possible that such a sudden increase in eotaxin-1 might impair cognitive abilities of the recipient. In this context, it is known that a considerable proportion of patients exhibit a temporary or even permanent decline in cognitive performance after major surgery, a phenomenon called postoperative cognitive dysfunction (POCD). The prevalence of POCD varies from 41% to 75% at 7 days to 18%–45% at 3 months postoperatively, depending on diagnosis criteria and surgery type studied (Monk and Price, 2011). The underlying mechanisms are not understood, however increasing evidence indicates that an enhanced inflammatory response might cause POCD. Indeed, it has been shown that surgery-induced release of several cytokines disrupts the BBB, leading to enhanced macrophage migration into the hippocampus, subsequent neuro-inflammation and POCD symptoms (Terrando et al., 2011). Of note, one study found that patients with an increased ratio of neutrophils to lymphocytes had a three-times higher probability to experience cognitive dysfunction after carotinoid surgery (Kayadibi et al., 2014). Moreover, intraoperative transfusion of ECs (>3 units) has been described as an independent risk factor for development of POCD (Zhu S.-H. et al., 2014). Considering eotaxin-1 as chemoattractant for neutrophils and its ability to pass the BBB, it is possible that high eotaxin-1 levels in FFP or EC from older donors might contribute to POCD in recipients.

While we were able to measure eotaxin-1 in all ECs and all but one FFP, it was detectable only in three of eight PCs with a low median expression of 10 pg/ml. In line with the age-dependent increase in eotaxin-1 found in FFP and EC, these three eotaxin-1 positive PCs have been donated by older donors (25–55) while it was not detectable in any sample from very young donors (<25 years; *n* = 4). A possible explanation for the fact that eotaxin-1 is almost absent in PCs is the pathogen inactivation process with amotosalen and UV light. This chemical substance binds nucleic acid with a high specificity, however, it has been shown that treatment of PC with amotosalen and UV light decreased expression of three proteins which are partly involved in chemokine receptor binding (Thiele et al., 2012). Further investigations should address the issue if pathogen inactivation influences the levels of eotaxin-1 and other chemokines/cytokines in PCs.

The source of eotaxin-1 in ECs has to be further investigated. Given that EC contain only 4% of residual plasma, the detected eotaxin-1 level cannot derive from this source. One possible explanation is eotaxin-1 binding to the small family of atypical chemokine receptors (ACKRs) such as duffy antigen receptor for chemokines (DARC), which is expressed on erythrocytes and able to bind eotaxin-1 (Nibbs and Graham, 2013). Although biological functions of chemokines are typically mediated by signaling through G protein-coupled chemokine receptors, they are also bound by ACKRs, which do not initiate classical signaling pathways after ligand binding, but rather act as chemokine storage depots or scavengers (Pruenster and Rot, 2006).

Concerns have been raised that longer storage time of blood products might negatively affect the recipient’s outcome after transfusion (Vandromme et al., 2009). In this context, it has been speculated that transfusion of longer stored blood products might lead to serious, even fatal adverse events in recipients (Lelubre et al., 2009). However, recent studies demonstrated that the storage time of blood products did neither increase the rate of adverse events after transfusion during birth-admission (Patterson et al., 2015), nor influence the mortality rates of critically ill adults (Lacroix et al., 2015) or patients undergoing complex cardiac surgery (Steiner et al., 2015). In line with this, we show that eotaxin-1 levels were independent of storage time of transfusion blood products, which ranged between 7 days and 76 days (FFP) and 4 days and 29 days (EC).

Some further questions could not be addressed within the present study. All used samples have been obtained from ready-to use blood products, which have been donated by volunteers after stringent, standardized medical examination. It thus can be assumed that all donors are in good health at the time of blood donation. Indeed, factors such as diseases, allergies, body weight, regular intake of drugs, alcohol consumption, dietary habits or others might influence eotaxin-1 levels. These questions have to be addressed separately. Moreover, during blood donation no data regarding the donor’s mental health or cognitive abilities have been collected. It is therefore not possible to correlate eotaxin-1 levels in donors with their cognitive capabilities. It also has to be considered that a direct impact of short-term eotaxin-1 exposure on neurogenesis and mental factors has only been assessed in mice so far. It is unclear whether a single transfusion of high-eotaxin-1 containing FFP or EC to recipients with very low levels will indeed change total eotaxin-1 levels in the recipient, if this elevation is transient or durable, and if it does indeed influence cognitive functions in humans. Future studies require a prospective study design to carefully resolve these questions.

## Author Contributions

JH performed all measurements and analyses and wrote the manuscript together with SJ and ZC. ML, SJ and HS designed the study. JH and ZC interpreted results. CD-P collected samples and data from donors. SJ and HS were responsible for the study.

## Conflict of Interest Statement

The authors declare that the research was conducted in the absence of any commercial or financial relationships that could be construed as a potential conflict of interest.

## Supplementary Material

Supplementary Figure 1 Summarized information on demographics of donors used for analysis of **(A)** fresh-frozen plasma (FFP), **(B)** erythrocyte concentrate (EC) and **(C)** platelet concentrate (PC).
