# Supplementary figures and images for: The “Aging Factor” Eotaxin-1 (CCL11) Is Detectable in Transfusion Blood Products and Increases with the Donor’s Age
Source: Front Aging Neurosci. 2017 Dec 1;9:402. doi: 10.3389/fnagi.2017.00402 (PMC5717008; doi:10.3389/fnagi.2017.00402)

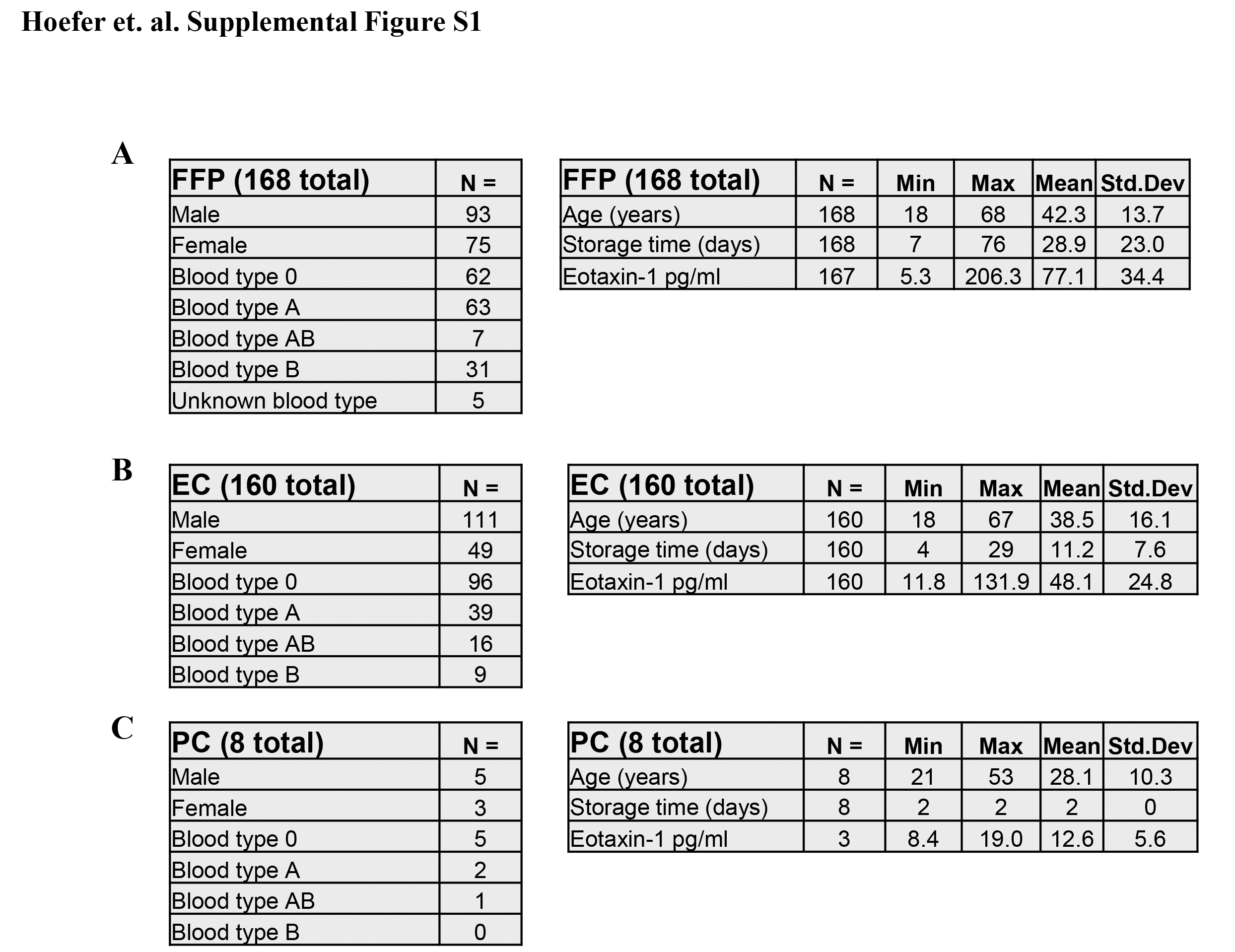

Supplement: Supplementary Figure 1 — Summarized information on demographics of donors used for analysis of (A) fresh-frozen plasma (FFP), (B) erythrocyte concentrate (EC) and (C) platelet concentrate (PC). [file Image_1.tif]
